# Ultrasound-guided superficial cervical plexus block for cancer-related clavicle pain

**DOI:** 10.1016/j.inpm.2022.100152

**Published:** 2022-10-14

**Authors:** Eliana Ege, Thomas Chai, Carlos J. Roldan, Billy K. Huh

**Affiliations:** a Baylor College of Medicine, 1 Baylor Plaza, Houston, TX, 77030, USA; b UT MD Anderson Cancer Center, 1400 Holcombe Blvd, Houston, TX, 77030, USA

**Keywords:** Cancer, Clavicle, Bone pain, Peripheral nerve block, Cervical plexus block, Ultrasound

## Abstract

Pain is one of the most feared conditions a cancer patient may face. Bone is a common site of metastasis in many malignancies, including breast, prostate, kidney, and lung cancer. Conventional therapy for tumor-related bone pain involves the use of opioids, non-steroidal anti-inflammatory drugs, and bisphosphonates. Palliative radiation therapy may be incorporated for refractory bone pain. We describe an innovative case of cancer-related clavicle pain successfully managed with a superficial cervical plexus block in an opioid-intolerant patient. Considering the lack of guidelines for pain interventions in this setting, such peripheral nerve blocks may be a useful adjunctive tool in refractory cancer pain management.

## 1 Introduction

Clavicular neoplasms are rare, comprising <1% of primary bone tumors and 2% of bone metastases [1,2]. As with any painful tumor, standard symptomatic management involves the use of opioid analgesics, co-analgesics, and adjuvants [3]. When proven refractory, bone pain can be further managed with palliative radiation therapy and various tumor ablation techniques [4,5]. Unfortunately, there is scarce literature on the management of neoplastic pain specifically involving the clavicle [6]. We report the novel and successful use of a superficial cervical plexus block in this setting.

## 2 Case presentation

A 58-year-old male with metastatic renal cell carcinoma presented to the pain clinic with a three-month history of left shoulder and clavicular pain. The patient described this pain as aching in quality, constantly a 9 out of 10 in severity using the Numerical Rating Scale (NRS), and aggravated by the movement of the left shoulder. He had a pre-existing history of bone metastases, including to the clavicle, as well as chronic bilateral shoulder pain. His symptoms had not responded to over-the-counter analgesics, such as ibuprofen and acetaminophen. He also wished to avoid the use of opioids, as tramadol and hydrocodone had previously caused intolerable side effects, including gastrointestinal disturbances and severe abdominal cramping. The patient had also undergone a left subacromial steroid injection by the orthopedic service with limited, transient relief.

**Fig. 1:**
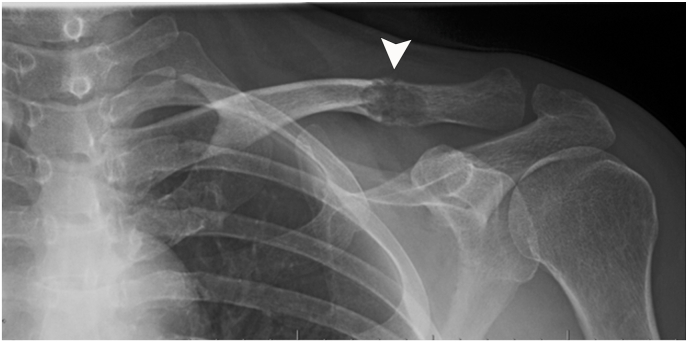
Radiograph of the left clavicle shows a lytic lesion (arrowhead) distally.

Physical examination revealed a palpable, tender mass in the left clavicle. The range of motion of the left shoulder was restricted actively and passively in all planes. Strength and sensation were preserved throughout the left upper extremity without any signs of neurovascular injury. Radiography of the left shoulder and chest demonstrated a mildly expansile lytic intramedullary lesion (measuring 25 mm ​× ​17 mm) in the distal clavicle, placing the bone at risk for an impending pathologic fracture (Fig. 1).

**Fig. 2:**
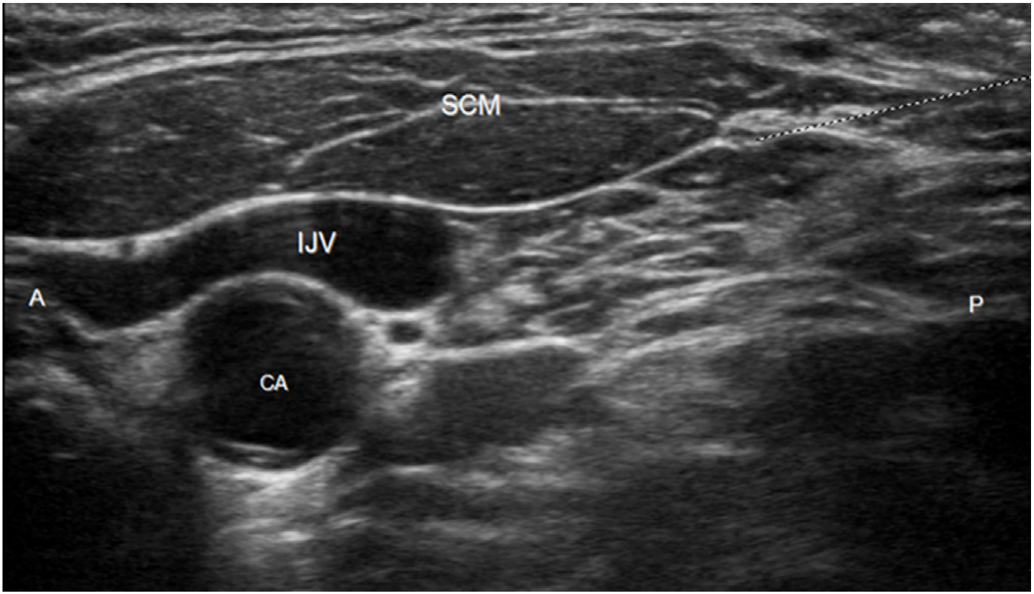
Ultrasound image of the superficial cervical plexus procedure showing the needle path for injection (dashed line). A ​= ​anterior; P ​= ​posterior, SCM ​= ​sternocleidomastoid, IJV ​= ​internal jugular vein, CA ​= ​carotid artery.

The patient was dispositioned to undergo cryoablation of the clavicular tumor, but treatment was delayed due to complications with care coordination and reimbursement. He inquired about temporizing measures to alleviate his severe clavicular pain in the meantime. Having exhausted conventional therapies, the patient was offered a left superficial cervical plexus block under ultrasound guidance, to which he consented. Using a high-frequency linear ultrasound transducer, a 5-mL mixture of 40 mg/mL triamcinolone and 0.25% bupivacaine was injected just deep to the midpoint of the posterior sternocleidomastoid muscle border (Fig. 2). The patient tolerated the procedure well and was observed for 30 minutes. No immediate post-injection complications were noted. The patient reported improvement from a pre-procedure pain score of 9/10 to 2/10 after the injection. He also noted cutaneous anesthesia over his upper shoulder and clavicle. His pain remained controlled without opioid analgesics or adjuvants until he was able to undergo cryoablation of the left clavicular lesion one week later.

## 3 Discussion

The cervical plexus is formed by the anterior rami of the upper cervical nerves, which give rise to superficial cutaneous and deeper muscular branches [7]. The superficial cervical plexus emerges behind the sternocleidomastoid muscle into four branches: the greater auricular nerve, lesser occipital nerve, transverse cervical nerve, and supraclavicular nerves [7]. These nerves innervate the skin and superficial structures of the anterolateral neck and shoulder [8]. The supraclavicular nerves are specifically thought to be involved in intrinsic bone innervation of the clavicle [8,9]. Cadaveric studies suggest that the supraclavicular nerves contribute to the cephalad and ventral aspects of the entire length of the clavicle, while the subclavian and lateral pectoral nerves supply the caudal and dorsal aspects [10].

Common indications for cervical plexus blocks include carotid endarterectomies, lymph node dissections, and superficial neck surgeries [7]. Recently, there has also been increasing interest in the use of regional blocks over general anesthesia for clavicle fractures, both in perioperative and emergency room settings [8,9,11]. The significant contribution of the supraclavicular nerves to the innervation of the clavicle renders them a key target for clavicle surgery. However, the use of a superficial cervical block for cancer-related clavicular pain has not been established in the literature. This block was offered to our patient based on the location of his tumor and the safety and efficacy of the procedure demonstrated in the aforementioned settings.

The only absolute contraindications to a superficial cervical plexus block are local infection or drug allergy [7]. Possible complications include infection, hematoma, local anesthetic toxicity, nerve injury, and inadvertent subarachnoid or epidural anesthesia [12]. However, this procedure has been shown to carry less risk of such events compared to deeper or combined blocks [12,13]. Moreover, ultrasound guidance may provide visualization of the spread of local anesthetic and monitoring of needle location to minimize complications [9].

The superficial cervical plexus block is often combined with a deep cervical plexus block, as well as an interscalene or supraclavicular brachial plexus block, depending on the indication [8,12,13]. The most efficacious type of block and composition of injectate are ongoing topics of debate [8,[12], [13], [14]]. The total volume of injectate administered during these procedures typically ranges from 5 to 15 mL. Due to the relatively superficial location of the superficial cervical plexus at the mid-point of the sternocleidomastoid, a total injection volume of 5 mL (1 mL of triamcinolone 40 mg/mL and 4 mL of bupivacaine 0.25%) was deemed sufficient in this case for adequate coverage of the targeted peripheral nerves while limiting the spread to more anterior or deeper cervical structures, such as the phrenic nerve. The steroid triamcinolone was included in the injectate due to its immediate membrane-stabilizing effect as well as subsequent anti-inflammatory and anti-nociceptive effects from inhibition of the phospholipid metabolism pathway to prostaglandin and leukotriene syntheses [15]. Prolongation of pain relief and reduction of pain intensity is expected with the addition of steroids for peripheral nerve blocks, although there is variability in outcomes [16]. In our case, the patient reported adequate pain relief for the week following the procedure, allowing him to avoid the use of opioid analgesics until cryoablation of the clavicular lesion by the Interventional Radiology team.

This case demonstrates not only a novel indication for this peripheral nerve block, but also an alternative treatment tool for pain management in the oncological population. Cancer pain, especially bone pain, necessitates a multimodal approach that combines physical, pharmacological, and interventional therapies. As in this instance, patients often experience delays and gaps in coordination of radiotherapy, tumor ablation, and other related care. Maintaining patient comfort throughout the process can therefore present a challenge, which can be compounded by refractory symptoms and opioid intolerance. The authors hope that this case may serve as an example for future investigation and optimization of interventions for pain relief in this challenging oncologic setting.

## 4 Conclusion

In this case, analgesic principles commonly utilized in head and neck surgeries were successfully applied to the management of severe cancer-related pain involving an uncommon site of metastasis. A superficial cervical plexus block performed under ultrasound guidance provided immediate pain relief without any complications in the setting of a clavicular tumor. As there are limited guidelines on pain management in this setting, the described approach may prove a useful addition to the treatment of severe cancer-related clavicle pain. Given the complexity of oncological treatment, peripheral nerve blocks such as the cervical plexus block should be further studied as adjunctive interventions for neoplastic pain, especially for patients with opioid intolerance.

## Informed consent

Informed consent was obtained from the patient whose case is discussed prior to the creation of this manuscript.

## Declaration of competing interest

The authors declare that they have no known competing financial interests or personal relationships that could have appeared to influence the work reported in this paper.
